# The importance of template choice in homology modeling. A 5-HT_6_R case study

**DOI:** 10.1186/1758-2946-5-S1-P8

**Published:** 2013-03-22

**Authors:** Krzysztof Rataj, Jagna Witek, Stefan Mordalski, Tomasz Kościółek, Andrzej J Bojarski

**Affiliations:** 1 Department of Medicinal Chemistry, Institute of Pharmacology, Polish Academy of Science, 12 Smętna street, 31-343 Kraków, Poland

## 

Over the years, homology modelling has grown into an important tool for biochemistry and pharmacology. It allows prediction of three-dimensional structure of proteins, which have not been resolved with empirical methods. The final outcome of such research is affected by many factors, the choice of template being a crucial one.

Current paradigm states, that proteins with the smallest evolutionary distance and thus, the highest identity/similarity, to the target, should achieve the highest performance. The goal of this research was to verify the credibility of this assumption when incorporating homology models into Virtual Screening protocol.

The target for this case study is 5-HT_6_R, which belongs to class A GPCR, and as a trans-membrane protein, is extremely hard to crystallize or solubilize. This makes standard protein structure assessment inexplicably difficult, however a few members of class A GPCR had their structure solved. 5-HT_6_R itself is involved in learning, memorizing and overall cognition processes, and is a target in anti-depression drug research [1,2].

This study comprised of homology modelling of 5-HT_6_R based on seven available GPCR templates (A2A, beta1, beta2, CXCR4, D3, H1, rhodopsin), and further verification of created models by means of ligand docking (Glide). The quality of generated structures was assessed in three subsequent steps, each consisting of different compounds sets for docking procedure. The final models were selected basing on the number of active ligands to decoys docked ratio. Interestingly, the templates used to construct the most successful models were not the evolutionarily closest ones, therefore putting the existing paradigm into question when it comes to VS application.
